# Supplementary material for: Resilient cooperators stabilize long-run cooperation in the finitely repeated Prisoner's Dilemma
Source: Nat Commun. 2017 Jan 13;8:13800. doi: 10.1038/ncomms13800 (PMC5241863; doi:10.1038/ncomms13800)
Supplement: Supplementary Information — Supplementary Figures, Supplementary Tables and Supplementary Methods [file ncomms13800-s1.pdf]

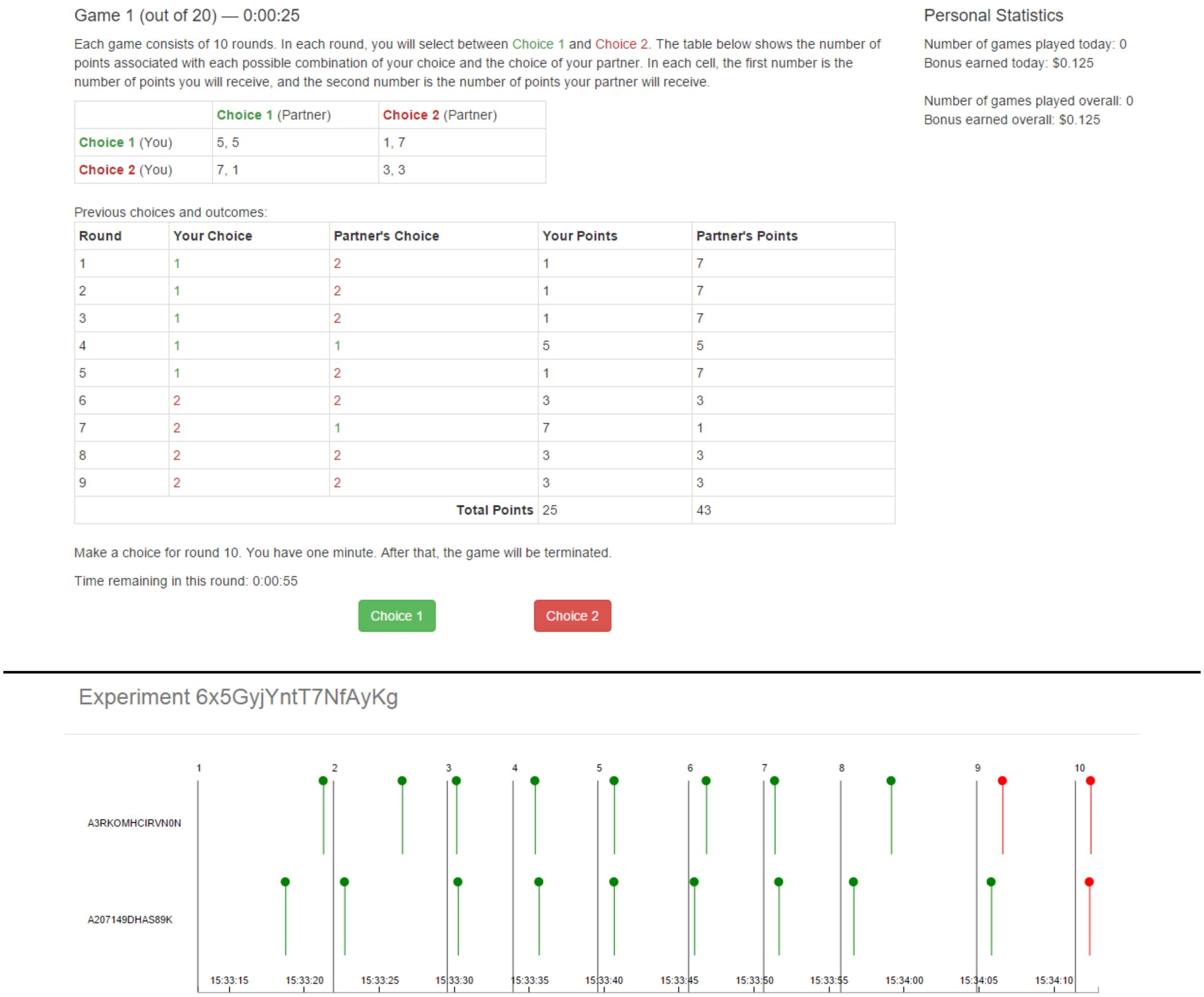

Supplementary Figure 1 – **(top)** Player's view of a single 10-round game, showing choices for each round as well as cumulative earnings and number of games. **(bottom)** Experimenter's view of player choices from an actual 10-round game, including actual decision times.

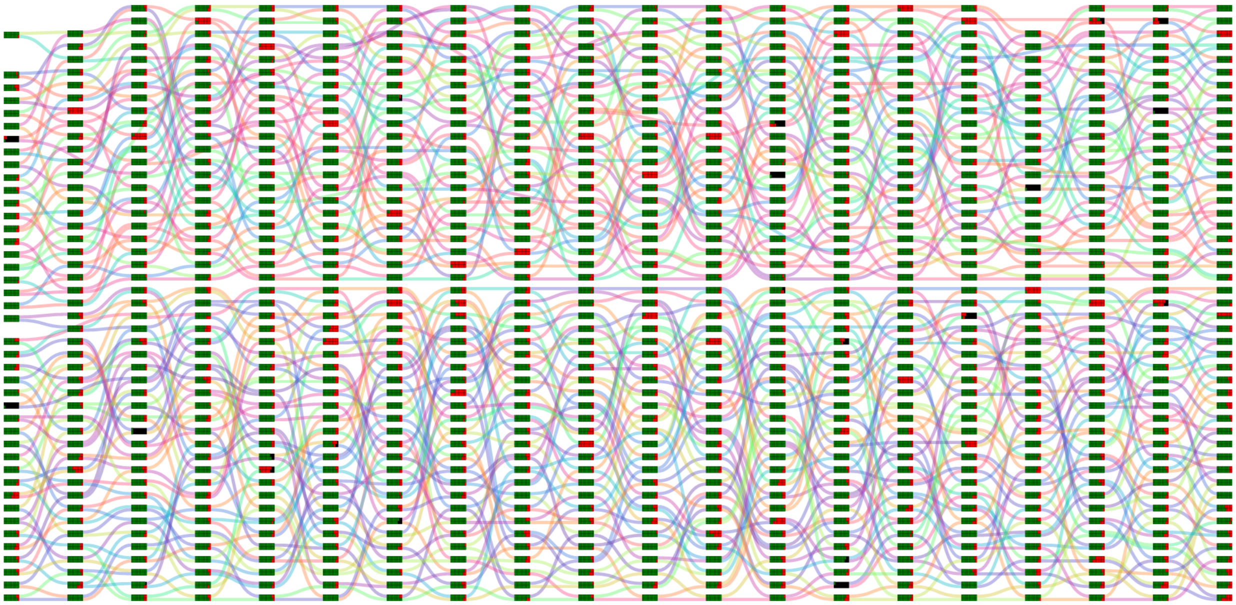

Supplementary Figure 2 – Visual depiction of one day of the experiment, showing about 100 players playing 50 simultaneous games, 20 times. (This shows the 15th day and the general pattern of behavior in the steady state.) Each rectangle represents one 10-round game and actions of two players therein where red pixels indicate defection and green pixels indicate cooperation. Colored bands denote an individual player, and show rematching between different partners.

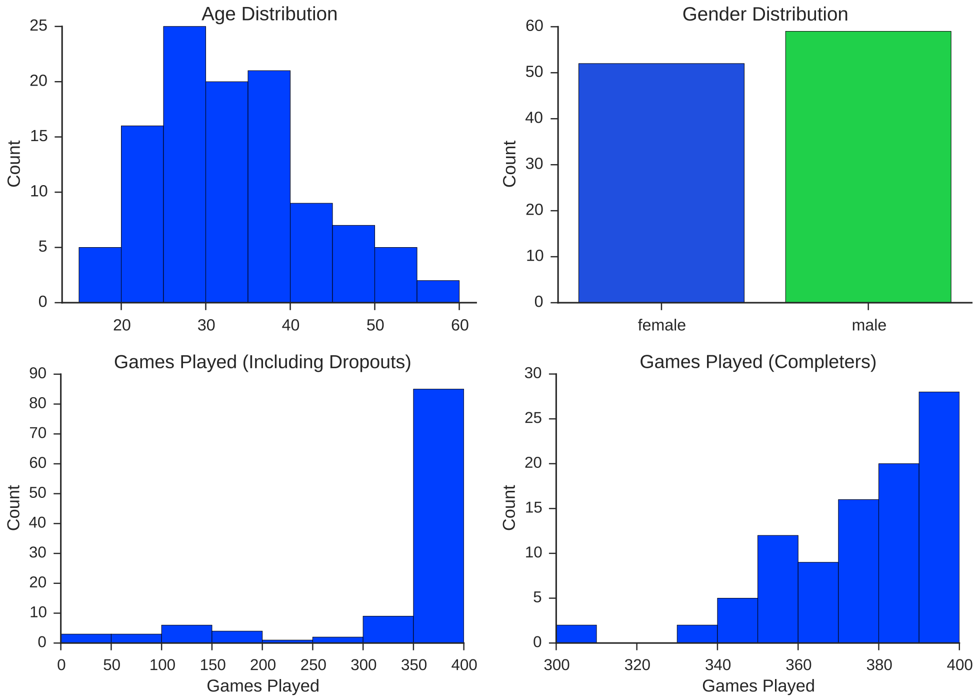

Supplementary Figure 3 – Demographics of the participant population based on their responses to the survey questions.

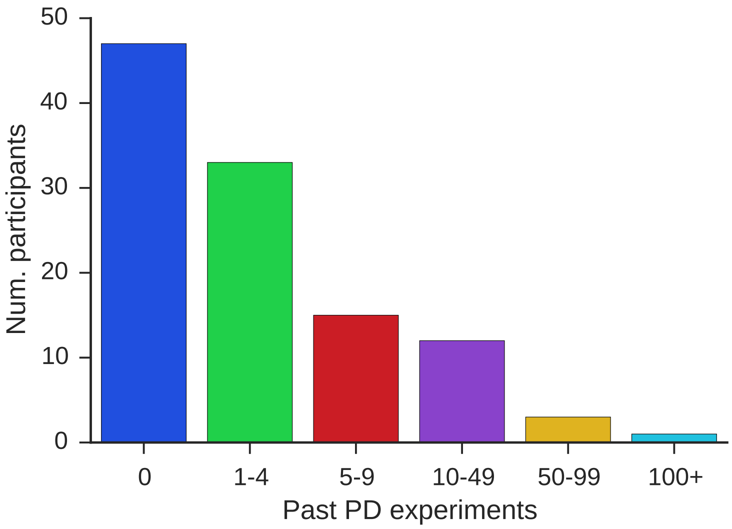

Supplementary Figure 4 – Responses given by players to the question: "The game you played in this study is known as *prisoner's dilemma*. **Before participating in this study**, on how many previous occasions had you played a similar game on Mechanical Turk?"

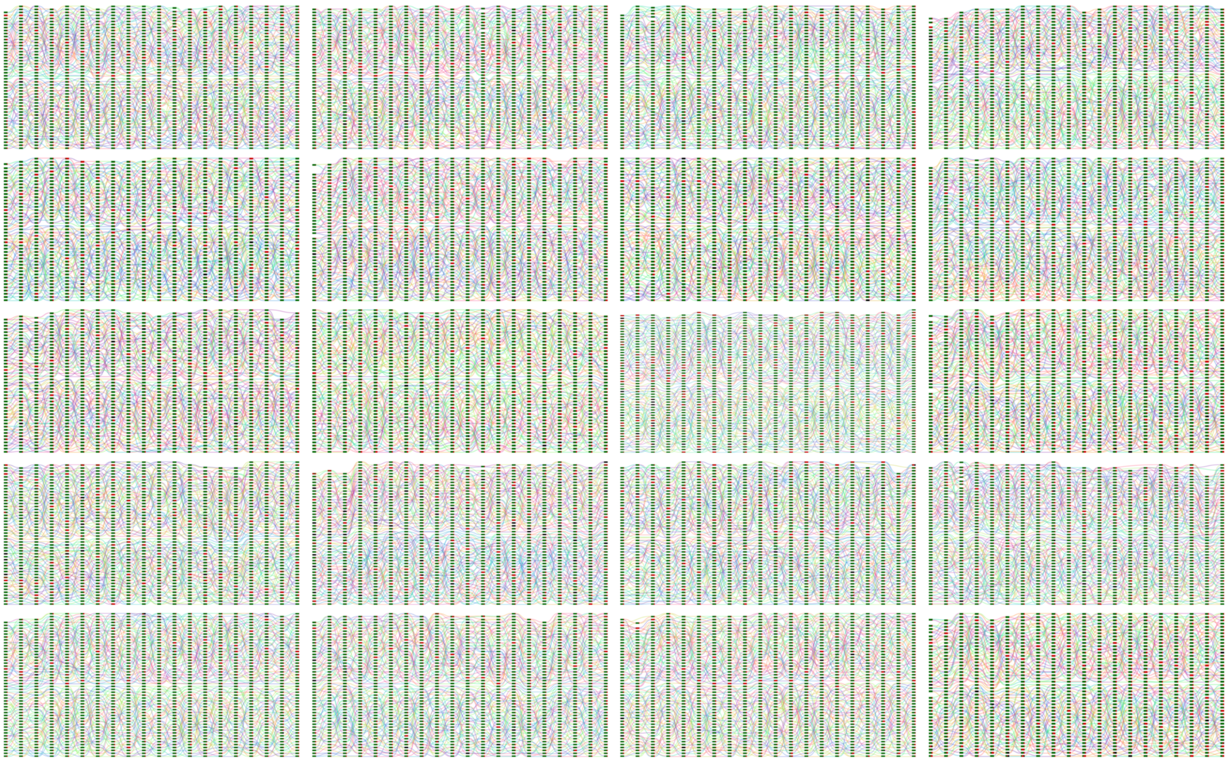

Supplementary Figure 5 – Visual depiction of all 20 days of the experiment

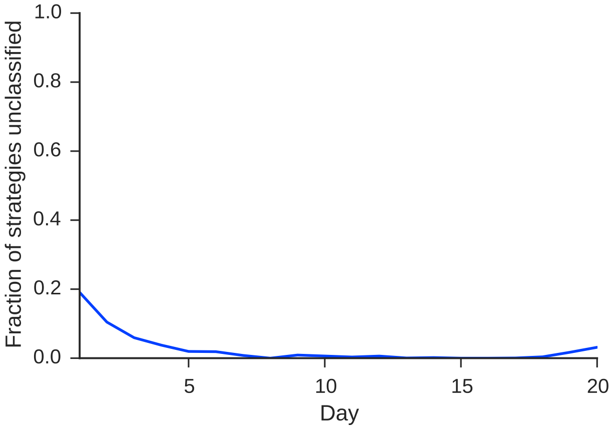

Supplementary Figure 6 – **Fraction of strategies classified as "other" (i.e. not CC or Threshold) by day.** As discussed in the main text, we classified all players in terms of both their observed behavior from the data and also their self-declared strategies. On day 1 almost 20% of strategies cannot be classified, as players switch back and forth between cooperate and defect during an initial learning period. However, we note that even this fraction falls to roughly 2% by day 5 and remains close to zero for the rest of the experiment.

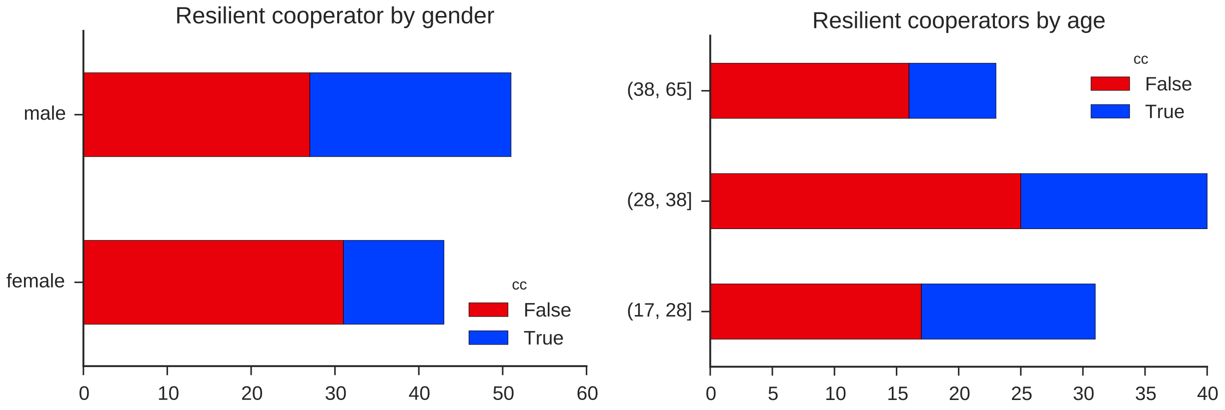

Supplementary Figure 7 – Rates of resilient cooperation by (A) gender and (B) age. For age we divide players into three age ranges. Using Fisher's exact test across all pairs of categories, no differences are significant at the 5% level.

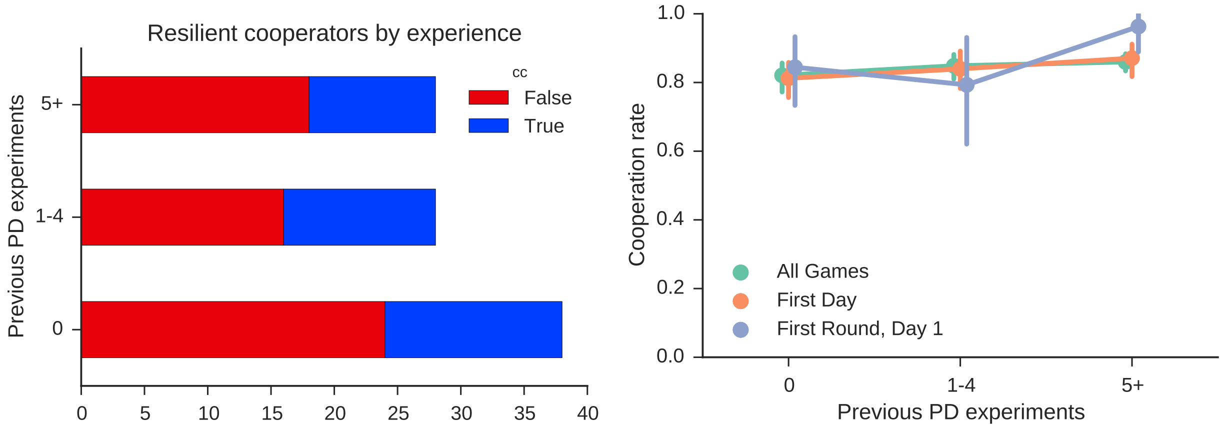

Supplementary Figure 8 – Effect of experience on cooperation. (A) Fraction of players classified as resilient cooperators based on past experience. The differences are not significant at the 5% level. (B) Average cooperation rates over the entire experiment, on the first day, and on the first round of the first game based on experience. In the case of overall cooperation and day 1) we conducted pairwise t-tests, whereas for round 1 of game 1 we used pairwise Fisher's exact tests (because when comparing play in a single round we have only one observation per person). In all cases the tests were insignificant at 5%, hence we conclude that behavior in our experiment does not vary significantly with self-reported experience.

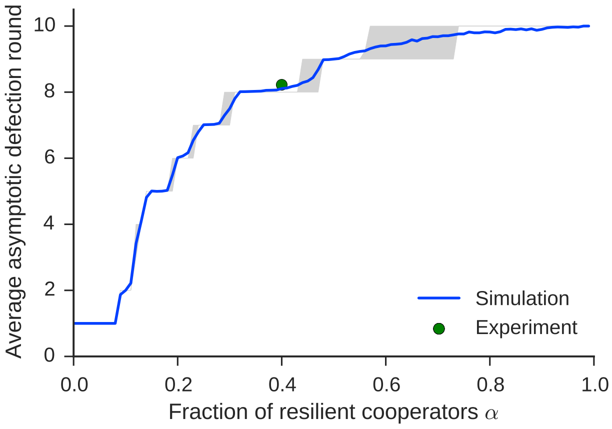

Supplementary Figure 9 – Interquartile range for asymptotic average round of first defection. For the vast majority of value of alpha, the IQR is scarcely larger than the line thickness (the blocky nature of the window is due to the integral nature of the round of first defection).

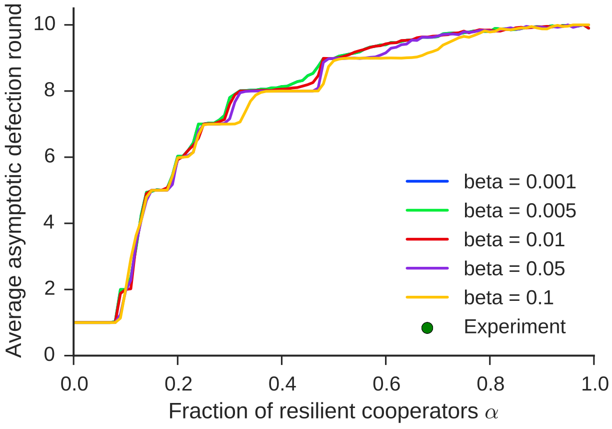

Supplementary Figure 10 – **Smoothed fictitious play model with different values of the smoothing parameter β.** Recall that $\beta \to 0$ implies that each agent deterministically selects the strategy with the highest expected utility, whereas $\beta \to \infty$ corresponds to agents making uniformly random choices of strategy (see Methods). Varying β by two orders of magnitude results in very similar predictions; thus we conclude that our simulation is not sensitive to this parameter over a wide range of values.Each line is the average of all rational agents in 10 sets of simulations with 100 agents each.

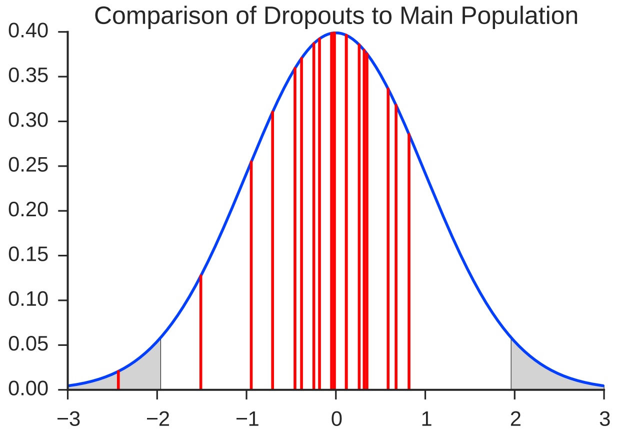

Supplementary Figure 11 – Daily cooperation rates of the 19 dropout players relative to the completed population over the same time period, shown as a standardized z-score. Shaded areas indicate the 5% most extreme values.

	Dependent variable:		
	average day cooperation		
	(1)	(2)	(3)
β_0 (intercept)	0.851***	0.852***	0.888***
	(0.006)	(0.007)	(0.026)
β_1 (day)	−0.001*	−0.001*	0.006***
	(0.001)	(0.001)	(0.002)
β_2 (dropout)		0.007	−0.031
		(0.022)	(0.049)
β_3 (dropout × day)		−0.003	−0.013*
		(0.003)	(0.008)
$\beta_{4,i}, \beta_{5,i}$ (player, player × day)			(219 parameters not shown)
Observations	1,960	1,960	1,960
R^2	0.001	0.003	0.845
Adjusted R^2	0.001	0.001	0.826
Residual Std. Error	0.134 (df = 1958)	0.134 (df = 1956)	0.056 (df = 1737)
F Statistic	2.891* (df = 1; 1958)	1.810 (df = 3; 1956)	42.775*** (df = 222; 1737)
Note:			*p<0.1; **p<0.05; ***p<0.01

Supplementary Table 1 – Regression results for models represented in equations 1, 2 and 3.

Day boundary	KS test statistic	KS p-value	MW test statistic	MW p-value
Day 1–2	0.119886***	0.000001	468179.0***	0.000192
Day 2–3	0.079265***	0.004222	452340.5*	0.069139
Day 3–4	0.116710***	0.000003	434550.5***	0.000139
Day 4–5	0.067663**	0.021901	472798.0	0.416333
Day 5–6	0.098592***	0.000130	435855.0***	0.000091
Day 6–7	0.080558***	0.003066	442348.5***	0.000078
Day 7–8	0.048479	0.192048	468674.5*	0.075663
Day 8–9	0.049679	0.179959	435583.0***	0.004242
Day 9–10	0.021636	0.978368	444000.5	0.330239
Day 10–11	0.031956	0.709569	430857.0**	0.025303
Day 11–12	0.026316	0.893396	437976.0	0.115994
Day 12–13	0.022215	0.973631	433163.0	0.330349
Day 13–14	0.017947	0.998458	410687.0	0.479973
Day 14–15	0.012800	0.999999	413930.0	0.451055
Day 15–16	0.017551	0.998843	411539.0	0.256666
Day 16–17	0.039885	0.459107	402405.0	0.208344
Day 17–18	0.046490	0.274213	400010.5	0.129383
Day 18–19	0.026689	0.899757	406589.0	0.358326
Day 19–20	0.042543	0.381455	390463.5*	0.071810

Note: $^{*}p<0.1$; $^{**}p<0.05$; $^{***}p<0.01$

Supplementary Table 2 – Kolmogorov-Smirnov and Mann-Whitney test statistics and p-values for changes in the distribution of round of first defection from one day to the next.

Supplementary Table 3 – Qualitative vs. quantitative analysis of strategies Classifications for all 94 players who completed the experiment as well as their responses to the two strategy-related questions in Section . **Inferred Strategy** denotes our analysis from the data, and **Qualitative Strategy** refers to our independent classification from the textual response. Note that in participants' responses, "Choice 1" (colored green) and "Choice 2" (colored red) refer to cooperate and defect, respectively, although they were not labeled as such in the experiment interface. We did explain cooperation and defection in debriefing, hence participants referred to these labels or colors interchangeably in their responses.

#	Inferred Strategy	Qualitative Strategy	**...did you follow any particular plan or strategy?**	**...did it change over the course of the experiment?**
1	CC	CC	"I always chose 1, hoping to gain the others trust and get the most points."	"I stopped pressing 2 when the other play pressed 2, and continued to press 1, hoping to gain their trust."
2	threshold	threshold	"I chose to cooperate for the first 8 rounds, and defect for the last 2, unless the other person defected before I did. . ."	"Sometimes I chose to defect on the 7th or 8th round, as others seemed to start defecting in round 9."
3	CC	CC	"I chose to cooperate each round but did play defensively and defected if my partner did so before the last round."	". . . I played the game as if I were face to face with my partner, how would I treat the person if I were looking at them. . . How about if I just went scorched earth from the start and defected, my partner would be caught off guard for a round or two but would then adjust. We would both lose, no gain for either of us. I chose to cooperate and only defect in defense."
4	threshold	threshold	"At first I wanted to select 1 every time, as it would lead to everyone getting the most money in the long run. Unfortunately, not everyone could see the big picture, so I had to change my strategy."	"Yes – after realizing too many other people in the group would choose option two near the end of the game, and them doing so would cause other people to do so as well – I started choosing choice 2 on round 9, choosing 1 in round ten if they chose 1, or 2 in round 10 if they chose 2."
5	CC	CC	"I mainly chose 1 so that me and my partner would both get a fair payout. If my partner ever chose 2, then I would choose 2 for all subsequent choices."	"If I felt that every person I was being partnered with was looking out for their own personal gain, then I would try to do the same. This only happened a few days for me."
6	threshold	threshold	"I settled on choosing 1 for the first 8 rounds and then selecting 2 for the remaining two rounds."	"My initial strategy was selecting all choice 1 for every game/round no matter what. My strategy changed because of non-cooperative partners. . . My strategies overall went from all choice 1 to then selecting choice 2 for the last couple of rounds. I almost started doing choice 2 at round 8 as well but didn't do that much at all, I didn't like it."
7	threshold	threshold	"I started off by trying to cooperate to the positive each time. . . it seemed that many people were willing to go along and do all 10 rounds positively. . . but as time wore on, it became evident that I was only being cheated over and over again in the final rounds. . . I started to see my daily bonus go down, and inevitably began defecting first on the last round, then in the 9th, and finally in the 8th round to counteract/preempt the defect that almost always was inevitably coming."	"Absolutely. For the first, maybe 10 games, I tried to go to the end, only defecting after someone else defected. . . I began to also defect in stages, (ie. first the 10th round, then into the 9th, and finally into the 8th). . ."
8	CC	CC	"I wanted to my partner and I to cooperate so we could both make good money."	"I was going to click all option 1's, but then the partner would choose option 2 about game 9 or 10, so I started doing the same some days, and some days I would pick option 1 every game no matter what just to see how much money I made."
9	threshold	N/A	"At the beginning I wanted to be fair and give each of me and the opposite player a fair chance at the money. . .Then I got "jaded" but the slower game players. . . if I suspected they were going to be one of the "problem" people, I would just start pressing the red one and not care about the money earned for myself. . ."	"Yes! Toward the middle of the month, I settled on 30 seconds to be at round 5 in a game was fast and if the player hit around that mark, I would be nice and give the green. If it took longer than that, they got the red. . ."

Continued on next page

Supplementary Table 3 – Qualitative vs. quantitative analysis of strategies Classifications for all 94 players who completed the experiment as well as their responses to the two strategy-related questions in Section . **Inferred Strategy** denotes our analysis from the data, and **Qualitative Strategy** refers to our independent classification from the textual response. Note that in participants' responses, "Choice 1" (colored green) and "Choice 2" (colored red) refer to cooperate and defect, respectively, although they were not labeled as such in the experiment interface. We did explain cooperation and defection in debriefing, hence participants referred to these labels or colors interchangeably in their responses.

#	Inferred Strategy	Qualitative Strategy	**...did you follow any particular plan or strategy?**	**...did it change over the course of the experiment?**
10	CC	CC	"I would always choose option 1 while my partner did the same, when they went to option 2 I followed. My goal was to make as many points for me as the other player. A few rounds I tried various strategies but always went back."	"For the most part no."
11	threshold	threshold	"I cooperated on every round before defecting in Round 8."	"In the first few games I would defect in round 10. Eventually I moved it up to round 9 as people caught on and then round 8..."
12	threshold	CC	"I played in the hopes that my partner would always choose Option 1. I saw it as such..."	"Somewhere around the middle, I did start choosing Option 2 in Round 9 (and even Round 8 in some cases)... by the end, I only chose Option 2 in response to a partner choosing Option 2. "
13	threshold	threshold	"For the first two days, I tried to defect on round 10 to get maximum money..."	"But I noticed that most players were doing this. So after day 2, I always defected on round 9... for the last two days, I started defecting on round 8, to get maximum points."
14	threshold	N/A	"At first I tried to be as fair as possible so both my partner and I could maximize our earnings."	"My strategy changed as more participant's became greedy... After a while, most players started defecting in the final round of each game."
15	threshold	threshold	"When I first started playing my strategy was to co-operate... shortly into the first game someone chose Choice 2 and therefore I only got 1 point and I was mad. For several sessions I decided I would just always make choice 2... Finally I decided to mostly cooperate and then around Round 7 to make the choice that would get me more points."	"... I changed from always making choice 2 to mostly cooperating is that I thought I would make more money."
16	threshold	threshold	"Each day I would start off trying to cooperate to the fullest. After a few rounds I would switch to using a 2 on either round 8 or 9... If the user continued to use 1 I would follow up with a 1 after the test to reward the others cooperation, knowing that the user would probably enter 2 for the remaining rounds..."	"Mostly the only change from above was moving from round 9 to 8 because 2 seemed to come up more often before round 10."
17	threshold	threshold	"I wanted to do what I could to get the most points for myself in the end. I chose choice 1 until the the 8th round, then I did choice 2."	"In the beginning, my strategy was to just stay with choice 1 throughout the whole game, however people started doing choice 2 so I did as well. "
18	threshold	threshold	"At first I decided to always play fair no matter what. I wanted things to be equal as much as possible. Eventually however, I changed my strategy a bit to protect myself... I decided that it would be best to defect after round eighteen..."	"At first i tried to use the first choice as much as possible, but after others defected sooner I also had too as well in order to keep things equal... I then decided that I needed to be proactive and assume that the person was going to defect after round eighteen... In a few cases it happened at round seventeen, but I did not change my strategy in this case even though it happened a few times. I felt that overall I would lose money if I changed my strategy to this round so I did not."
19	threshold	threshold	"I chose option 1 on round 1-8, and option 2 on 9 & 10 of each game."	"Yes, I started out choosing option 2 only on the last round of each game, but later started choosing option 2 on rounds 9 & 10 when the other players began choosing option 2 more often on the last round."

Continued on next page

Supplementary Table 3 – Qualitative vs. quantitative analysis of strategies Classifications for all 94 players who completed the experiment as well as their responses to the two strategy-related questions in Section . **Inferred Strategy** denotes our analysis from the data, and **Qualitative Strategy** refers to our independent classification from the textual response. Note that in participants' responses, "Choice 1" (colored green) and "Choice 2" (colored red) refer to cooperate and defect, respectively, although they were not labeled as such in the experiment interface. We did explain cooperation and defection in debriefing, hence participants referred to these labels or colors interchangeably in their responses.

#	Inferred Strategy	Qualitative Strategy	**...did you follow any particular plan or strategy?**	**...did it change over the course of the experiment?**
20	threshold	threshold	"Pretty much stuck with the plan to choose '1' throughout most of the game, then switch to '2' towards the end."	"In the beginning, I'd alternated between 1 and 2 for whatever reason, but eventually figured that one the opponent had stuck with 2, there wasn't any real reason for me to choose 1 unless I wanted to give him/her free points."
21	threshold	threshold	"I tried to maximize my payout, assuming my partner would be doing the same, choosing one was beneficial to us both."	"i began to defect in the later rounds when I noticed that others were doing do in order to minimize the loss."
22	CC	CC	"I just tried to be as far as possible and select 1 every time. I did this until the other player selected 2 and then I would start choosing that."	"It didn't really change over the course of the study. It just depended on what the other person did."
23	CC	CC	"My goal was to come away with the most money. It seems clear to me (Despite what the debrief says) that the economically rational thing to do is to cooperate. If we are all defecting, we will all earn much less then if we are all cooperating..."	"This was my strategy for the entire game."
24	CC	CC	"if all players cooperate at all times, benefit is maximized for the group... I was determined to choose the cooperative choice every round until and unless my partner chose to defect. Even as players began the slip toward defecting in round 10, then round 9, then round 8 or earlier as the social creep began to set in, I chose to never be the first to stab someone in the back..."	"My strategy never changed. I went with choice 1 every game, every round, until my partner chose choice 2, at which point I switched to choice 2 in all future rounds to prevent further losses."
25	CC	CC	"I felt that I should always cooperate until the other person defected. I knew that if everyone started just defecting, people would just start defecting earlier and earlier until the bonus was abysmal."	"Nothing changed. I stuck with that strategy the entire time."
26	threshold	threshold	"i tried to keep the scores even, eventually trying to sneak in a few more once the other person thought i would pick the same number again"	"i altered the round in which i chose to change numbers"
27	threshold	threshold	"I would try to cooperate (action 1), but then realized most people would pick option 2 at the end, so I would typically select 2 for the last two rounds."	"I originally just picked all 1s as I thought everyone getting 50 per round was better than getting less than 50."
28	CC	CC	"I chose to cooperate unless the other person chose the defect at which point I chose to defect too for the rest of the game."	"I started by cooperating unless the other player defected but I changed to defecting on the last round... I returned to only defecting if the other player defected first to be fair to other players that cooperate."
29	threshold	CC	"I wanted to choose 1 as much as possible, to maximize everyone's gain. I was willing to go 1 every single time if the other players were willing to as well. If there were too many 2's though, I would adapt..."	"Nope, I stuck with the same philosophy the whole time. On a bad day I might be more willing to press 2 though."
30	threshold	threshold	"I just kept hitting choice 2 because it seemed that I would win each round and always get the max bonus."	"no it was the same for all month of the survey"
31	threshold	threshold	"I chose the Choice 1 for most of the rounds and then Option 2 for last couple rounds because it made most points. If my partner switched to Option 2 before I anticipated doing so, then I switched too..."	"No, my strategy did not change."

Continued on next page

Supplementary Table 3 – Qualitative vs. quantitative analysis of strategies Classifications for all 94 players who completed the experiment as well as their responses to the two strategy-related questions in Section . **Inferred Strategy** denotes our analysis from the data, and **Qualitative Strategy** refers to our independent classification from the textual response. Note that in participants' responses, "Choice 1" (colored green) and "Choice 2" (colored red) refer to cooperate and defect, respectively, although they were not labeled as such in the experiment interface. We did explain cooperation and defection in debriefing, hence participants referred to these labels or colors interchangeably in their responses.

#	Inferred Strategy	Qualitative Strategy	**...did you follow any particular plan or strategy?**	**...did it change over the course of the experiment?**
32	CC	CC	"I chose to go with Option 1 as the default, until an opposite player would choose Option 2..."	"Over time, I would sometimes choose Option 2 in the last selection, since a lot of people were doing the same..."
33	CC	CC	"I would always choose to cooperate until my partner defected, at which point, I would defect."	"My strategy did not change. "
34	threshold	N/A	"It depended on my mood and how the game was going for me that day. If I had some players who "2 bombed" everything I would play more selfishly to catch up and prevent not losing out more to others."	"I think I became less cooperative as it progressed and less worried about others especially if my earning that day were low due to other's actions or being left out of rounds."
35	threshold	threshold	"Well my plan each time was to cooperate... once my partner continued to defect I felt I had to also to protect my own interest... So to keep myself from being on the losing end many rounds I automatically defected because I expected that was what my partner would do."	"I tried to follow a strategy of being cooperative each time and would have preferred to that throughout. But because my partners were not I felt forced to defect too. "
36	threshold	N/A	"trying to get highest number"	"no"
37	CC	CC	"I tried to always choose option 1 and give us both the best earning opportunity unless the other person did not co-operate, in which case I would select choice 2."	"My strategy initially was to choose option 1 most of the time and then choose option 2 for the last two rounds but eventually I did option 1 the entire time unless the other person wasn't co-operating. "
38	threshold	CC	"I wanted to make sure it was fair, so I chose 1 always, unless someone chose 2. If I was being shorted by people picking 2 near the end, I'd chose 2 near the end against others and try to even it out by picking 1 if they didn't also pick 2 next round."	"My strategy stayed the same, pick 1 unless given a reason not to. Maximize earnings for both parties."
39	threshold	threshold	"As it soon became apparent that all players understood cooperation will maximize payoff, I went along with the flow and selected option 1 up until the last round. If players appeared to be "playing" me (eg varying the times between choices) I would be more likely to select choice 2 in an earlier round..."	"After i saw that timing variation in clicking, or just super-fast clicking, often coincided with players becoming uncooperative early, I tried to get ahead of them by clicking choice 2 earlier than planned."
40	CC	threshold	"At first, I chose for both me and the other player to get five points each round. I then thought hmm, maybe I can make a little more, so the last round I would make it so I would get 7 and the other would get 1. But eventually others started doing the same thing... So I then started trying to get 7 on the round 9 and then 3 on round 10. But I had a theory that this would overall cause a decrease in amount made... So I figured I might as well try to get others to adopt a better strategy... and more people started going with 5 and 5 all the way through. But alot were still trying to get that 7 at the end, so I chose towards the end to choose option 2 on round nine so I would either get 3 or 7 points. If I got 7, I would give the other player 7 on the next round assuming they chose option 2 on round ten. If I got 3, I would continue with option 2 so we would both 3..."	"explained above"

Continued on next page

Supplementary Table 3 – Qualitative vs. quantitative analysis of strategies Classifications for all 94 players who completed the experiment as well as their responses to the two strategy-related questions in Section . **Inferred Strategy** denotes our analysis from the data, and **Qualitative Strategy** refers to our independent classification from the textual response. Note that in participants' responses, "Choice 1" (colored green) and "Choice 2" (colored red) refer to cooperate and defect, respectively, although they were not labeled as such in the experiment interface. We did explain cooperation and defection in debriefing, hence participants referred to these labels or colors interchangeably in their responses.

#	Inferred Strategy	Qualitative Strategy	**...did you follow any particular plan or strategy?**	**...did it change over the course of the experiment?**
41	threshold	threshold	"Most of the time, I tried to select the first option... However, once in a while I would pick option 2 near the end to try to get the extra 6 points for a better payoff. I didn't want to do that too often though because I felt guilty as it wasn't fair to the other player."	"Sometimes I did change strategies to try to get back the points I may have lost because the other player "got me" with the second option more than once, before I changed to option 2. I would try to keep the options fair by choosing option 1, but sometimes the other player would start off with option 2 and I would try to get them back to option 1..."
42	threshold	threshold	"I began by finding the general idea of how the group might play on the first day and seeing that mostly everyone would go 1's throughout except for maybe on 10 but not often at all. As the experiment progressed more people would start going 2 on 10... So as more people went 2 on 10 I decided that I would at least try to protect myself from getting 46 total points by splitting 2's on 10 by going 2 on 9 as well... However this became a trend and typically people would now always go 2 on rounds 9 and 10. I felt going beyond this was idiotic because in the end to continue in this fashion you are jeopardizing the whole groups pay. I felt comfortable either going 1 for all or 2 on 9 or 8 sometimes..."	"See above answer."
43	threshold	threshold	"I started off trying to be fair and play equally. Knowing that we were playing the same participants every day, I tried to learn what patterns others were playing so I could adjust my play to benefit me but still be fair."	"... When others would choose to take it all the last round, I started doing that also, choosing option 2 for tenth round only... Then I kept getting others who would start choosing 2 for the 9th & 10th rounds, so I did as well. For most of the month, I played choice 1 for the first 8 rounds then choice 2 for the last two rounds... This last 4-5 games, I ended up choosing option 2 for round 7 as well. I changed when others started taking more in earlier rounds."
44	threshold	threshold	"Early on in the study and early on during most individual days, I went with Choice 1 because I was attempting to keep things equal for both people. As time went on, I started to defect in order to play defense for myself... I started defecting around round 9 for most of the study and around round 8 at some points toward the end of the study."	"Yes, my strategy changed from keeping things equal to defecting earlier and earlier to protect my earnings. It was not a fool proof strategy..."
45	CC	CC	"I chose to mostly cooperate - I wanted to maximize payment for myself and the other player. Going into the games, I figured I would mostly cooperate and then deflect as others deflected."	"It stayed mostly the same, I cooperated more than I thought I would. I guess I kind of hoped it would encourage others to continue to cooperate more as well."
46	CC	CC	"I tried to get the best outcome for me and the other person, which was by picking option one. However, if they tried to pull the best option in their favor, I would switch to option two so I wouldn't be left out in earnings."	"No, I kept the same strategy the entire time."
47	threshold	threshold	"to cooperate as much as possible, until the end for my advantage"	"when others caught on, I had to start switching sooner"

Continued on next page

Supplementary Table 3 – Qualitative vs. quantitative analysis of strategies Classifications for all 94 players who completed the experiment as well as their responses to the two strategy-related questions in Section . **Inferred Strategy** denotes our analysis from the data, and **Qualitative Strategy** refers to our independent classification from the textual response. Note that in participants' responses, "Choice 1" (colored green) and "Choice 2" (colored red) refer to cooperate and defect, respectively, although they were not labeled as such in the experiment interface. We did explain cooperation and defection in debriefing, hence participants referred to these labels or colors interchangeably in their responses.

#	Inferred Strategy	Qualitative Strategy	**...did you follow any particular plan or strategy?**	**...did it change over the course of the experiment?**
48	threshold	threshold	"Well, I figured out that the only way to gain any extra money was to hit choice 2 at the very end (10th round)... you're only going to be able to get that 2 in there once before they start hitting 2 also... So, I chose one every time except for the last round, UNLESS my partner starting choosing 2 earlier, then I chose 2 as well to protect myself."	"I fiddled around with it a little bit the first week, but I settled on my strategy after that and kept it going straight through."
49	CC	CC	"Cooperating with the other player so I could at least gain some points."	"For the most part, it changed toward round 8 and 9 and 10. Players were more inclined to give themselves more versus sharing."
50	threshold	threshold	"I eventually settled on choosing 2 for the final three rounds. Toward the end of the study, many people were choosing 2 on the final two rounds. I chose to press 2 one round before most people..."	"My original strategy was to only press 2 on the tenth round when most people were choosing all 1. As the game progressed, more and more people were choosing 2 for the final round and I changed my strategy to compensate. I then started choosing 2 for the final two rounds in order to maximize my points. Toward the end of the study many other people were utilizing the same strategy, so I started pressing 2 for the final three rounds."
51	CC	CC	"I made Choice 1 until I saw the other person making Choice 2, and then I matched them for the remaining rounds."	"In the first day of the game, I made Choice 2 at the end of one of the rounds to get extra money. I felt really guilty about doing this and decided it wasn't worth the emotional turmoil for me! So I continued by always making Choice 1 and only ever reacting to other greedy players once they made Choice 2."
52	threshold	threshold	"I thought choosing choice 1 for the first and majority of the beginning rounds would allow both me and the other person to earn more stably. Towards the ending rounds, I would often choose choice 2 at rounds such as 8, 9, or 10 to avoid ending with less points... If I missed out on a round, I would have also chosen choice 2 more often to make up for the points."	"I don't think it really changed over the course of the study. There were slight adjustments depending on how previous players acted but I don't think they were significant."
53	threshold	N/A	"When i began i initially chose to defect, and would do so for a while. But then i realized that was not a fair strategy and reversed when i would defect."	"It solidified over a couple of days and stuck to it as it was reaping the most benefits."
54	CC	threshold	"I started out by trying to be cooperative... I was concerned that the more I defected, the more mistrust would seep into the game and the worse everyone would do (though my concern was with my own results, not others')... I was willing to lose a few pennies each game if it meant people cooperated for 8 or 9 rounds at least... However, after weeks of feeling like I was being taken advantage of by people who defected in round 8 or 9, I started to defect more... the change in motivation here was to protect myself, not to be greedy and steal from others. Had I KNOWN others would cooperate, I would have done that..."	"... in the last week I was tired of being taken advantage of in round 8 or 9, and so I started defecting in round 9 or 10 in a lot of the games. I didn't WANT to punish someone who was playing cooperatively, but I was very tired of being defected on when I was willing to be cooperative. My desire to be cooperative eventually was overcome by my desire not to be a sucker."
55	threshold	threshold	"None in particular. I started out choosing 1, seen others choosing 2 on Round 9 and started doing that."	"I was choosing one a lot more in the end."

Continued on next page

Supplementary Table 3 – Qualitative vs. quantitative analysis of strategies Classifications for all 94 players who completed the experiment as well as their responses to the two strategy-related questions in Section . **Inferred Strategy** denotes our analysis from the data, and **Qualitative Strategy** refers to our independent classification from the textual response. Note that in participants' responses, "Choice 1" (colored green) and "Choice 2" (colored red) refer to cooperate and defect, respectively, although they were not labeled as such in the experiment interface. We did explain cooperation and defection in debriefing, hence participants referred to these labels or colors interchangeably in their responses.

#	Inferred Strategy	Qualitative Strategy	**...did you follow any particular plan or strategy?**	**...did it change over the course of the experiment?**
56	CC	CC	"I always chose to cooperate, except when my partner chose to defect I also chose to defect for the remaining games."	"no, the same strategies"
57	threshold	threshold	" I learned it was best to choose option 1 for the 1st 8 or 9 and then spring for choice 2 at the end, normally the last 1 or 2 games. That way they thought I was being cooperative and then I'd surprise them sometimes. . ."	"Yes. When I 1st started playing I always went for what I thought would earn me a higher bonus (choice 2) right off the bat. I soon learned it was best to save that option for the last 1 or 2 games in each round and instead play choice 1 for the 1st 8 or 9 and then spring for choice 2 at the end. "
58	threshold	threshold	"My goal every time was to maximize my earnings. I also wanted the other person to do well but there was no way for me to know what they were going to do. Anytime I chose to defect it was only to protect myself, not to "steal" money or take advantage of the other player. . . Some players defected earlier and that was always a terrible choice, especially against me as I would then defect every round after no matter what."	"At first I always wanted to cooperate but I then realized that defected in round 10 had no consequences. But after a while I kept getting burned in round 9 so I then started to defect in that round as well. Then the same thing kept happening in round 8 so again I started to defect. . ."
59	threshold	threshold	"I would try and chose the best option for the most points for both but at times i would change the last 2 rounds"	"I foloowed the strategy that i would chose option 1 for the first 8 rounds and the last 2 rounds i would chsoe option 2"
60	CC	CC	"The best option for both parties was to cooperate, thus I chose option 1 to start with and only switched when the other party chose option 2. . ."	"No, again, best bet for both parties was option 1, saw no reason to change unless the other player chose option 2."
61	threshold	threshold	"I selected choice 1 for the first 7 rounds, then chose choice 2 for the 8th round. If my opponent also selected choice 2 for the 8th round, then I would select choice 2 for the remaining rounds because I could see that they were trying to beat me. If my opponent chose choice 1 for the 8th round, I would choose choice 1 for the 9th round to let them win that round for not trying to trick me and then possibly even out again. (7/1, 1/7). I would choose choice 2 for round 10."	"I originally selected choice 1 for all rounds until I saw that some of my opponents switched up, which made me end up with less."
62	CC	CC	"I wanted to continue to cooperate as long as possible. That would maximize the common payout"	"Every now and then I defected, but I didn't really have a strategy other than doing it late in a round"
63	threshold	threshold	"...I started then selecting 1 all the way up until round 10. After a few days people caught on, so I had to start doing "2" in round 9. . ."	"It changed a bit as I described previously, but after a week, the strategy was basically the same. "
64	CC	CC	"I tried to cooperate until I grew frustrated with people not doing so. I didn't see a point in choosing 2 before round 9. I switched back and forth depending on how it was going and how angry I was getting with people."	"I didn't really change my strategy over the course of the study, I would change it during each day depending on how I was feeling about how the game was going. "
65	threshold	CC	"Ideally I wanted both my partner and I to pick choice 1 every time. Once I saw someone pick choice 2, I followed so I would still get 3 points."	"Same strategy"
66	threshold	threshold	"I tried to cooperate, but after seeing people defect (most often in the last 2 rounds) I started defecting myself in the rounds 9 and 10 most of the time."	"I started off just cooperating all the time as I figured it was fairest to try to give all of us the same payoff. Eventually I started defecting in the last two rounds as I saw more players doing this and it maximized my earnings."

Continued on next page

Supplementary Table 3 – Qualitative vs. quantitative analysis of strategies Classifications for all 94 players who completed the experiment as well as their responses to the two strategy-related questions in Section . **Inferred Strategy** denotes our analysis from the data, and **Qualitative Strategy** refers to our independent classification from the textual response. Note that in participants' responses, "Choice 1" (colored green) and "Choice 2" (colored red) refer to cooperate and defect, respectively, although they were not labeled as such in the experiment interface. We did explain cooperation and defection in debriefing, hence participants referred to these labels or colors interchangeably in their responses.

#	Inferred Strategy	Qualitative Strategy	**...did you follow any particular plan or strategy?**	**...did it change over the course of the experiment?**
67	threshold	threshold	"I choose to cooperate for the first nine rounds and then defect on the tenth. If the other user defected earlier, I would defect as well."	"My strategy stayed the same throughout the study."
68	threshold	threshold	"i pasically always tried to pick choice 2 number 7 or 8"	"no i tried to keep that"
69	CC	CC	"I had a strategy I used throughout most of the games, which was in trusting the other player to choose the same options as I did, option 1... I played option 1 during all the rounds, all the time, unless I saw the other player choose option 2 in round 9, or earlier, in which case I would switch to option 2 as well."	"I followed my strategy closely throughout the course of the study, and only noticed I had to change toward the end, when other players started choosing option 2 as early as in round 7..."
70	threshold	CC	"First week... I picked option 2 for choice 10, then 2 for choice 9 then 2 for choice 8, all to maximize my points. I realized this was counter productive towards the end, to maximize both mine and other other person' income, it was more beneficial to cooperate. Indeed towards the end of the trial, more people started playing straight 1 choices for the whole 10 rounds with me."	"I started picking option 2 for the 10th choice, then I would do it earlier, because other people started copying this strategy. Halfway through I decided to play straight 1 choices unless the other player would pick a choice 2 at any point."
71	threshold	threshold	"I would stick with option 1 until round 8, then switch to 2 as I realized most players change at round 10."	"Every now and then, I tested changing a move earlier or one move later. I eventually changed from defection on round 8 to round 9 for optimal points."
72	CC	CC	"Knowing that it was long term, it made sense to begin with a fair split, choice 1, as a good will gesture and hope that both players could continue to do so rather than sacrifice the other player's money for my own gain."	"... My strategy of fairness became punitive when the other player selected choice 2 early on or repeatedly. I mulled my choices over and sometimes mixed up choices in order to trip up the other person... I mostly selected Choice 1 without really thinking about it."
73	CC	CC	"i wanted to maximize profits for both players."	"Not really."
74	CC	CC	"...In this situation, it is theoretically better, on average, for everyone to choose to cooperate... the most rational choice would be to choose to cooperate for the first 19 rounds, and the defect on the 20th... As can be expected, though, many players chose to defect in the last few rounds... So my default choices for the first 19 rounds were determined by rationality; no one would choose to cooperated in a later round if I had defected previously. The choice for round 20 was determined out of idealism and universal reasoning. If a player I faced chose to defect, I would generally defect for the rest of the round. "	"For the most part, I don't think my strategy changed at all. I made a small number of misclicks, but my decision making was fairly consistent throughout."
75	threshold	threshold	"...Ultimately, my strategy was to use choice 1 for 9 rounds and switch to choice 2 in the final round. Unless the other player switched to choice 2 earlier in which case I played choice 2 from that point onwards. I decided not to add to the collective distrust by switching earlier than that."	"The first day I was just using choice 1 all the time. And for a few games, so did my partners. Quickly people started switching on the last round, and it got irritating losing 2 cents, so I did the same..."
76	threshold	threshold	"i went for overall highest points. generally i'd cooperate until the 9th or 10th round, then defect. that would increase my points."	"i defected more on days where it seemed like other players were defecting more. otherwise i cooperated because it got me the most overall points."

Continued on next page

Supplementary Table 3 – **Qualitative vs. quantitative analysis of strategies** Classifications for all 94 players who completed the experiment as well as their responses to the two strategy-related questions in Section . **Inferred Strategy** denotes our analysis from the data, and **Qualitative Strategy** refers to our independent classification from the textual response. Note that in participants' responses, "Choice 1" (colored green) and "Choice 2" (colored red) refer to cooperate and defect, respectively, although they were not labeled as such in the experiment interface. We did explain cooperation and defection in debriefing, hence participants referred to these labels or colors interchangeably in their responses.

#	Inferred Strategy	Qualitative Strategy	**...did you follow any particular plan or strategy?**	**...did it change over the course of the experiment?**
77	threshold	threshold	"I tended to select option 1 every round until the other player chose option 2 for 2 or 3 consecutive games. after that I defected in round 9 to try to get a jump on the other player."	"It did change significantly. I figured it was in both players' best interest to choose option 1 in every round. I tried to stick to that for a while, but found that people didn't see it the same way... In the end, I would choose #1 all the way until people started defecting in round 9 or 10. At that point I would defect in round 9 and if the other player chose option 1 in round 9, I would choose 1 in round 10 so their points and mine would be equal."
78	threshold	threshold	"I tried to cooperate on the first nine rounds, assuming the person cooperated in previous rounds. Then, I would not cooperate on the last round - I felt this was the best way to play."	"Eventually, I chose more and more to not cooperate on the second to last round, as many people were doing the same to me... However, I am not sure if this actually made me more money, but if I continued to cooperate when others did not, I feel like it would have cost me money."
79	threshold	threshold	"I started off early in the month with the strategy of trying to cooperate until the very last minute (round 9 or 10 or so is when I would choose option 2). However, I gradually noticed more and more people going for the 9th and 10th rounds, so I decided to usually choose option 2 directly after the 7th round..."	"I think my only strategy was to try and predict what the person was going to choose. I was thinking about if previous "
80	CC	CC	"When I played my first game I decided to try and maximize my returns, but then felt like it was a jerk thing to do... So I switched to a strategy of only defecting if the other player did it first."	"Same as the other question, I ended up using the strategy to only defect if the other play defected before me. "
81	CC	CC	"I tried to be fair and honest when dealing with my partner but there were times where they would cooperate. I took it upon myself to not cooperate with my partner in later rounds to get back at them for taking my generosity for advantage."	"My strategy changed depending on which partner I was matched with. I would cooperate until my partner decided to defect."
82	threshold	threshold	"At first I started out cooperating, but I noticed that, after a while, people began choosing to defect near the end of each game... I started defecting near the end of each game as well."	"First I cooperated, then I started to defect around the ninth round of each game (sometimes the eighth, to shake things up). I would sometimes... start cooperating again through entire games, until someone would defect on me..."
83	threshold	threshold	"I settled on choosing 1 up until the 9th turn. I chose 2 for the 9th and 10th turn."	"In the beginning I chose a variety of numbers in each turn. Then I started choosing 2 on the 10th turn. I changed this strategy because I noticed more and more people choosing 2 before the 10th turn. "
84	CC	CC	"I always cooperated until the other person didn't."	"I stuck to the same strategy throughout..."
85	CC	CC	"I pretty much always went with choice one... If everyone with with choice two, then everyone actually earns far less than they do if they cooperate..."	"The strategy didn't really change for me. I did choice one as long as the other person did. Once they did choice two, I immediately did it to counter them..."
86	CC	CC	"I chose to cooperate until my partner did not. After they didn't, I didn't anymore either so as to not reward them for choosing 2."	"I didn't change mine at all. I knew that choosing 1 more would benefit us both, so I chose that every time until my partner chose 2"

Continued on next page

Supplementary Table 3 – Qualitative vs. quantitative analysis of strategies Classifications for all 94 players who completed the experiment as well as their responses to the two strategy-related questions in Section . **Inferred Strategy** denotes our analysis from the data, and **Qualitative Strategy** refers to our independent classification from the textual response. Note that in participants' responses, "Choice 1" (colored green) and "Choice 2" (colored red) refer to cooperate and defect, respectively, although they were not labeled as such in the experiment interface. We did explain cooperation and defection in debriefing, hence participants referred to these labels or colors interchangeably in their responses.

#	Inferred Strategy	Qualitative Strategy	**...did you follow any particular plan or strategy?**	**...did it change over the course of the experiment?**
87	threshold	threshold	"I wanted to make the most money possible."	"I knew as soon as someone chose to screw the other player over the rest of the 10 choices would result in less money for both of us. Which meant to make any money at all you had to cooperate until the 9th or 10th turn. If you decided to stop cooperating before that you'd end up with less money in the end."
88	CC	CC	"I would choose 1 unless my partner decided to take advantage and choose 2, then I'd choose 2..."	"Not really. I did try a few different things, but stayed with the same strategy overall."
89	threshold	threshold	"For the first few games, I would just choose Option 1 for all ten rounds, usually giving both players 25 points. However, after a few games, I would pick Option 2 for the tenth round, usually gaining me an extra couple of points. By the eleventh game, I would choose Option 2 for the ninth and tenth round."	"It really only changed when my partner would start off with Option 2, forcing ME to choose option 2 as well, and gaining us fewer points. I would also change if my partner picked option 2 earlier than I anticipated."
90	CC	CC	"My original strategy was to wait to see what the other player was going to do. I was expecting the other players to maybe choose 2 in the middle of the game. I was going to chose 2 and see if they would go back to 1."	"It changed the first day. With a few exceptions, the other players weren't defecting until the 9th or 10th rounds. I did the math. At the current point value, it's only beneficial if you defect at the 10th game... So I waited for the other person to 2 me. I kept telling myself that I was going to 2 the person in the 10th round, but rarely did. I felt that if they trusted me all the way to the 10th round to not 2 them, I wasn't going to break their trust."
91	CC	CC	"My plan and strategy is always to be fair and cooperative unless given reason not to."	"No it didn't change."
92	threshold	threshold	"... I knew if I selected choice 2 early on, it would make my partner retaliate and potentially I would end up with a minimal bonus... I would choose 1 until the 8th choice of each round... by waiting until the 8th turn I minimized my partners retaliation. When my partner noticed my pattern I would change, instead of choice 2 on the 8th turn I would choose 2 on the 7th..."	"I primarily stuck with my strategy as mentioned above, however, a few times my partner would choose 2 as soon as the game started, in return I would choose 2 and both of us ended with a low bonus. That only happened a few times and I would choose 2 on the 5th turn in the next game following that. This made me feel as if I had some control of how I would receive a bonus."
93	threshold	threshold	"I usually chose option one, since that would garner the most points. If you picked option 2 right away, the partner would not pick option 1. I usually picked option 2 for choice 9 and 10, because sometimes the person would pick one and I would get 7 points... I noticed I got the most points when I mostly cooperated..."	"No, I pretty much did the same strategy for the whole thing, choice one for all except the last few choices. Mostly you could not trust the other person would pick choice one for the last two, so you had to pick choice two or end up with fewer points."
94	CC	threshold	"Depending on how I felt that day I would either defect in round 9 or round 10. As the study went on went with round 9 more often as was the rest of the group I believe."	"At first I only defected in round 10 but after the first week I was getting screwed over a lot so I started defecting in round 9."

Supplementary Methods

Experiment Instructions

Welcome This HIT will grant you a qualification for a month-long research study consisting of 20 sessions where you will play a game with other workers. Each session will start at the same time every weekday (Monday through Friday) and take about 20-30 minutes, and the sessions will take place over the course of about 4 weeks. We expect the experiment to start the week of August 3, 2015 and run at least through August 28, 2015.

If you choose to participate in the study, **you will need to be present for every session**. If you miss more than 2 sessions, you will no longer be allowed to participate. **Please only agree to participate if you can make a commitment to be present for all 4 weeks.**

Because this is a research project, we also ask that you please **do not discuss the study in online forums or chat rooms** while participating.

In this HIT, we will ask about your availability and schedule the sessions at a time when the most workers are available. If you agree to participate, we will contact you when the study begins and specify the time that you should arrive on each day. Since the study involves **interacting with other workers in real-time**, it is important that on each day you **arrive on time and start the HIT immediately**.

Once you read these instructions, you will see a short quiz that tests your understanding of the rules of the game (as described below). If you fail the quiz twice, you will not be able to participate in the study.

The Game In each session you will play a sequence of up to 20 games where you will be randomly paired with another player.

Each game consists of a sequence of 10 rounds. Each round, you have a choice between two different actions (Choice 1 and Choice 2). You will have one minute to make your decision. After both you and your partner make a decision, you will each receive a certain number of points. This table shows how many points you and your partner will earn based on your choices:

	**Choice 1** (Partner)	**Choice 2** (Partner)
**Choice 1** (You)	5, 5	1, 7
**Choice 2** (You)	7, 1	3, 3

In each cell, the first number is how many points you will receive, and the second how many points your partner will receive. In other words:

- If both you and your partner select Choice 1, you each earn 5 points.
- If you select Choice 1 and your partner selects Choice 2, you earn 1 point and your partner earns 7 points.
- If you select Choice 2 and your partner selects Choice 1, you earn 7 points and your partner earns 1 point.
- If both you and your partner select Choice 2, you each earn 3 points.

Each game ends after 10 rounds, and you will be randomly matched with another partner for the next game. Once you have completed 20 games (possibly fewer, given the availability of other players), you will be able to submit the HIT.

You will then return the next day to complete another session, and so on for 20 sessions over the course of 4 weeks.

Payment You will receive a base payment of 10 cents for each of the 20 sessions. For each session, you will also earn a bonus based on the total number of points you receive. We will convert your score to dollars at the rate of 2 points = 1 cent. We expect this will allow you to earn a minimum of **$9.00 per hour**. You will receive an **additional bonus payment of $20** if you are still participating at the end of the 20 sessions. This bonus will be at least 20% of your earnings.

In total, you will be able to earn a minimum of $80.00 (including the additional bonus payment) through fully participating in this study.

Exit Survey

Thanks for participating in this research study! Please answer the following questions.

- What is your age?
- What is your gender? [Male, Female]
- What is your occupation?
- What is your location? (US ZIP code or Canadian postal code)?
- The game you played in this study is known as *prisoner's dilemma*. **Before participating in this study**, on how many previous occasions had you played a similar game on Mechanical Turk? [0, 1–4, 5–9, 10–49, 50–99, 100+]
- In some experiments, such as this one, participants play many individual games of prisoner's dilemma. For instance, in this study, you played about 20 games each day. **Before participating in this study**, how many individual games of prisoner's dilemma had you played on Mechanical Turk? [0, 1–4, 5–9, 10–49, 50–99, 100+]
- Another variant of this game is known as a *public goods game*. In this game, players voluntarily contribute some fraction of their wealth to a common pool that is then shared equally among all participants. **Before participating in this study**, on how many previous occasions had you played a public goods game on Mechanical Turk? [0, 1–4, 5–9, 10–49, 50–99, 100+]
- **Before participating in this study**, how many individual games of public goods had you played on Mechanical Turk? [0, 1–4, 5–9, 10–49, 50–99, 100+]
- In choosing your actions in each game, what particular plan or strategy did you settle on, if any? Please describe it in your own words.
- If you followed a strategy, did it change over the course of the study? What were your previous strategies? When and why did they change?
- What strategies, if any, did you notice other players using?
- Did the strategies of other players appear to change over time?
- Did you learn anything about **yourself** during this study?
- Did you learn anything about **other people** during this study?
- Do you feel like the people you played with developed any social norms or standards as a group, whether implicitly or explicitly? If so, describe them.
- **Imagine the study had continued for another 20 days.** What do you think would have changed, if anything?
- Did you discuss this study with other workers (either in person, in forums, or in chat rooms) while playing? If so, what did you find most intriguing to talk about?
- Do you think the compensation was fair?
- Would you be interested in playing similar games on Mechanical Turk in the future? How might we make it easier for you to participate over a long period of time?
- Would you be interested in playing similar games on Mechanical Turk in the future? How might we make it easier for you to participate over a long period of time?
- Do you have any other comments or feedback for us regarding this study?
